# Health hazard allowance for Nursing professionals: A reflective analysis under the principle of human dignity

**DOI:** 10.1590/1518-8345.5397.3498

**Published:** 2021-11-19

**Authors:** Rita de Cassia Ezaias, Maria Helena Palucci Marziale, Jair Aparecido Cardoso

**Affiliations:** 1 Empresa Municipal de Desenvolvimento Urbano e Rural de Bauru, Setor Jurídico, Bauru, SP, Brazil.; 2 Universidade de São Paulo, Escola de Enfermagem de Ribeirão Preto, PAHO/WHO Collaborating Centre for Nursing Research Development, Ribeirão Preto, SP, Brazil.; 3 Universidade de São Paulo, Faculdade de Direito de Ribeirão Preto, Ribeirão Preto, SP, Brazil.

**Keywords:** Occupational Health Nursing, Occupational Risks, Containment of Biohazards, Labor Legislation, Nursing, Risk Factors, Salud Laboral, Riesgos Laborales, Contención de Riesgos Biológicos, Legislación Laboral, Enfermería, Fatores de Riesgo, Saúde do Trabalhador, Riscos Ocupacionais, Contenção de Riscos Biológicos, Legislação Trabalhista, Enfermagem, Fatores de Risco

## Abstract

**Objective:**

to discuss the classification of the health hazard allowance due to exposure to biological agents attributed to Nursing professionals, based on legal and occupational parameters supported on the principle of human dignity.

**Method:**

an original reflection study with theoretical analysis on legislation, jurisprudence and Occupational Health focused on the biological risks, health hazard and rights of Brazilian workers. The discussions were based on the current legislation and on scientific evidence.

**Results:**

the classification of the health hazard allowance due to exposure to biological agents attributed to Nursing professionals is not in line with the factual situation experienced by them.

**Conclusion:**

it becomes necessary to broaden the discussion on the subject matter and to review the effective and fair compensation of Nursing professionals due to exposure to potentially contaminated biological agents in their work environments, given that the health hazard allowance is a worker’s right and is based on human dignity.

## Abstract (es)

**Objetivo:**

discutir la clasificación del adicional por insalubridad por exposición a agentes biológicos asignada a los profesionales de enfermería, con base en parámetros legales y ocupacionales fundados en el principio de la dignidad humana.

**Método:**

estudio original de reflexión con análisis teórico sobre legislación, jurisprudencia y Salud Ocupacional centrado en los riesgos biológicos, las condiciones insalubres y los derechos de los trabajadores brasileños. Las discusiones se basaron en la legislación actual y la evidencia científica.

**Resultados:**

la clasificación del adicional por insalubridad por exposición a agentes biológicos asignada a los profesionales de enfermería no se corresponde con la situación fáctica que experimentan.

**Conclusión:**

es necesario ampliar la discusión sobre el tema y revisar la indemnización efectiva y justa para los profesionales de enfermería por exposición a agentes biológicos potencialmente contaminados en sus ambientes de trabajo, dado que el adicional por insalubridad es un derecho del trabajador y se basa en la dignidad humana.

## Abstract (pt)

**Objetivo:**

discutir a classificação do adicional de insalubridade por exposição aos agentes biológicos atribuída aos profissionais de enfermagem, a partir de parâmetros jurídicos e ocupacionais apoiados no princípio da dignidade humana.

**Método:**

estudo original de reflexão com análise teórica na legislação, jurisprudência e Saúde Ocupacional com enfoque nos riscos biológicos, insalubridade e direitos dos trabalhadores brasileiros. As discussões foram embasadas na legislação vigente e em evidências científicas.

**Resultados:**

a classificação do adicional de insalubridade por exposição aos agentes biológicos, atribuída aos profissionais de enfermagem não está em consonância com a situação fática vivenciada por eles.

**Conclusão:**

faz-se necessário ampliar a discussão sobre o assunto e rever a efetiva e justa indenização dos profissionais de enfermagem por exposição aos agentes biológicos potencialmente contaminados em seus ambientes laborais, haja vista que o adicional de insalubridade é um direito do trabalhador e tem como fundamento a dignidade da pessoa humana.

## Introduction

Health care is fundamental and indispensable to protect human dignity. Nursing professionals are of utmost importance in this context, due to their decisive and proactive role in relation to the identification of health care measures, as well as health promotion and protection in the different dimensions and stages of human life. Due to this variety of tasks, they need to have dignified conditions to safely exercise their professional practices^(1)^.

However, practice of the profession is carried out in environments that involve biological, chemical, physical and psychosocial occupational risks, anti-ergonomic situations and, in many institutions, inadequate and unsafe working conditions are observed^(2)^. In this context, it is noteworthy that the provision of decent, safe and secure work environments is a prerogative that integrates the Sustainable Development Goals (SDGs) of the United Nations 2030 Agenda^(3)^.

In view of this complex scenario that involves health professionals in Brazil, it is necessary to discuss the classification of the activity by exposure to biological agents to assess whether there is an unfair setting, based on the premise that the legal standard would not be in line with the factual situation experienced. Without discrediting any professional activity and escaping from the scientific and academic purpose herein pursued, an extremely important question arises: If all other professions subjected to health hazards are entitled to a technical evaluation of the biological classification and other characteristics to indicate the existence of unhealthy conditions and the framing level, why only Nursing professionals would be subjected to the legal classification? Wouldn’t this provision be restrictive and unfair? Wouldn’t it be a deficient legal protection that encounters principle-based obstacles, or would it be a mistaken discrimination?

The work environment and the time of exposure to biological agents of these professionals are not properly defined, in view of the outdated Annex 14 of Regulatory Standard (*Norma Regulamentadora,* NR) No. 15, of Ordinance 3,214/78 of the Ministry of Labor and Employment (*Ministério do Trabalho e Emprego*, MTE)^(4)^. Thus, the environmental conditions and biological risks show that the compensation resulting from the health hazard allowance is not effective in the sense of protecting life and, therefore, does not meet the axiological and teleological principles of the standard, in addition to offending the principle of human dignity that aims at protecting workers’ integrity, which is a constitutional right.

The *motto* here is not to emphasize the logic of monetization as a solution to the problem of health hazard or a way of protecting life. On the contrary, health in the work environment is advocated, as well as prioritization of the life protection mechanisms, through effective safety and health standards in the work environment. When these solutions are ineffective, not because of negligence by the service taker, but because of the working conditions themselves, ultimately protective, they are paid for, as if this paid for the value of life, which is not true. It is considered that this short-sighted view does not agree with the principles of defending human dignity, nor with the human and fundamental rights established in the Federal Constitution (FC) in force. Added to the assertion is the fact that the pecuniary solution paid is unfair, which is even more aggravated when, for hidden reasons, payment of these amounts does not occur correctly.

It is to be emphasized that human dignity is a supreme value that attracts the content of all fundamental rights. It is a commitment to absolute and unrestricted respect for the identity and integrity of every human being, as a subject of law. In this bias, the worker’s physical integrity must be protected. And if workers are exposed to an unhealthy environment, which cannot be mitigated or excluded by the use of Personal Protective Equipment (PPE), they must be guaranteed the right to a fair compensation^*^ for the harms their health can suffer from exposure to biological agents, as the ultimate reason of the corollary of protecting human dignity.

Thus, the objective of the study evidences the need to discuss the classification of the health hazard allowance due to exposure to biological agents attributed to Nursing professionals, based on legal and occupational parameters supported on the principle of human dignity.

## Method

The scope of this study was a reflection with theoretical analysis on legislation, jurisprudence and Occupational Health supported on the principle of human dignity, focused on the biological risks, health hazard conditions and workers’ rights. The discussions were based on the current legislation and on scientific evidence published in the national and international literature. The elements presented for reflection were the historical overview of biological risk in the Nursing context, the health hazard allowance for Nursing professionals and the principle of human dignity, as a constitutional foundation for the health hazard allowance.

The study was carried out between June 2020 and April 2021 by analyzing the current legislation on the theme and its historical basis, as well as the legal principle guiding the health hazard allowance provided for in Annex 14 of NR 15^(4)^, which defines the unhealthy professional activities which can harm workers’ health and quality of life over time, with emphasis on the biological risk to which Nursing professionals are exposed and whose health hazard due to biological risk is qualitatively classified as of maximum degree (activities that include operations of permanent contact with patients in isolation due to infectious diseases and their non-sterile use objects) and as of medium degree (operations in permanent contact with patients, animals or infectious materials in places for the health care of the people, hospitals, laboratories and health units, among others).

Furthermore, texts in the Nursing area were analyzed with a historical and current focus on exposure to contaminating biological agents. The authors fully read scientific articles, theses and dissertations, books and legal documents - reports on health hazards in Nursing published on the classification of the health hazard allowance due to exposure to biological agents attributed to Nursing professionals available in databases and in published institutional websites, with no date limits established.

## Results and Discussion

### The historical overview of biological risk in the Nursing context

The NR regarding the classification of the health hazard allowance for biological agents, in its Annex 14 of NR 15^(4)^, was established in 1978, in a technical context, in which patients with infectious diseases were assisted in hospitals typically destined for isolation, in order to prevent transmission of such diseases.

Originally, the hospitals served as a shelter for pilgrims, the poor, the disabled and the sick. The services were performed by laypeople, mainly religious, not being the exclusive locus for the medical practice. Care of the sick itself used to be performed by family members in their homes. The purpose of hospitals as care locus for the sick came only with the development of capitalism. The first ones were built in London and later expanded to other locations, to reduce mortality caused by major epidemics through access to health services, and to redefine their function, in order to recover the workforce^(5)^. At that time, the hospital hygiene conditions were precarious and the Nursing practice was rudimentary.

The use of new interventions and technologies resulting from scientific advances such as asepsis, antisepsis, disinfection, sterilization, antibiotic therapy and different forms of isolation, as well as reports of contamination in professionals, led to the adoption of isolations and precautions again, to prevent transmission of pathogenic microorganisms, both for patients and for professionals^(5)^.

In the contagion theory, the prevailing concept was that infectious diseases multiplied through touch or contact of their bodies, which is currently known as “direct contact”. Such theory stimulated practices to control and restrict individuals, culminating in the institutionalization of quarantine^(6)^. To corroborate this idea, we recall the institutions for the maintenance of leprosy colonies that proliferated as a result of the leprosy epidemic, from 1920 onwards, in several Brazilian states. The program to combat the disease included compulsory isolation in several places; however, this program weakened the patient’s social and family relationships.

From 1958 onwards, the isolation extinction process occurred due to the effectiveness of medications. In 1962, there was the prohibition of compulsory hospitalization, although this continued until mid-1980s. Mistaken notions of contagion brought about harms in the health area for decades^(6)^.

In this way, it is worth mentioning here the origin of the word “miasma”, which derives from the Greek and originally meant “stain” or “pollution” for a sin of offense to the Gods. Later, the term designated putrefying airs and atmospheres, associating them as causative agents of diseases^(7)^. In the nineteenth century, it was understood that diseases were caused by atmospheric impurities resulting from the decomposition of animals and plants, humidity, garbage and housing close to each other and crowded^(7-8)^. Therefore, the notions of contagion, miasmas and associated practices precede the scientific theories about the spread of epidemics and infectious diseases^(8)^. The germ theory overcame these notions, developing a modern concept of transmission of infectious diseases, showing that these diseases occur through the infectious transmission of microorganisms or biological agents, through specific pathways.

The definition of how these pathogens are transmitted from one individual to another guides the formulation of preventive and rational discourses that break with the dissemination of fear and irrational behaviors associated with the old notions of contagion and miasmas^(6)^. According to FUNDACENTRO^(6)^, Annex 14 of NR 15 is outdated and not in line with the scientific advances, which makes the provision therein contained obsolete.

Scientific evidence shows that the focus is not on the infectious diseases or associated biological agents, but on a set of factors that include aspects related to the work environments and activities, in workers, users/patients, animals and potentially contagious materials. It is considered that the current approach involuntarily and subtly stimulates fear and irrational attitudes associated with the concepts of contagion, placing the risks in patients, workers and workplaces, which can foster discrimination and prejudice of the health services.

Nursing professionals’ exposure to biological agents is not the same as at the time when Annex 14 of NR 15^(4)^ was approved, since there are no longer typically isolation hospitals, such as sanatoriums for the treatment of patients with tuberculosis; these professionals are currently exposed to biological risks in different areas of health care institutions, then in permanent contact with potentially contaminated materials and people with infectious diseases.

From this perspective, it is worth noting here that the epidemiological transition process encompasses three basic changes: the “(...) replacement of communicable diseases by non-communicable diseases and external causes; shifting the burden of morbidity and mortality from younger groups to older groups and transformation of a situation in which mortality predominates to another in which morbidity is dominant”^(9)^. There is no way to measure the worker’s exposure time to biological risk because this depends on the understanding of the Nursing work process.

In a literature review study, a number of researchers analyzed the available scientific evidence on the microorganisms that colonize health workers and their association with antimicrobial resistance; in the ten-year period from 2007 to 2017; the evidence revealed that *Staphylococcus aureus* is the main colonizing bacteria in health workers, among which potential resistance to beta-lactam antibiotics, commonly used in hospitals, was found^(10)^.

Although the aforementioned bacteria is part of the normal microbiota of any human being, health professionals present an alteration in their individual microbiota, giving rise to resistance to antibiotics. Therefore, they are constantly exposed to multi-resistant microorganisms that damage their health, due to activities and work environments^(10)^.

For these reasons, it is imperative that the Standard points out the work activities of these professionals and their respective environments, which include hospitals, pre-hospital care service (*Serviço de Atendimento Móvel de Urgência*, SAMU), Basic Health Units, Emergency Care Units, urgency and emergency services and other health facilities. The normative setting of the health hazard percentage, without taking these variants into account, is characterized by an evident discrimination against those who carry out their activities in the health area, in flagrant disrespect of their dignity. The discriminatory aspect must also be highlighted, as no other professional activity has such restriction.

Thus, it is evidenced that the parameters used by the Standard are not in line with the factual situation of Nursing professionals and offend the principle of human dignity, as they do not represent an effective and adequate compensation, regarding the ultimate reason (*ratio*) and not fostering of monetization.

### Health hazard allowance for Nursing professionals

The Occupational Health and Safety Standards aim at reducing or eliminating occupational risks, thus protecting human health, even with all the risks. It is important to stress that it is up to the employer to comply with and enforce them.

The idea of eliminating risks must prevail in the work environment, meeting the principles of precaution and prevention, simultaneously. However, if this risk is not eliminated, the responsible person must legally respond.

Thus, the scope of this research is limited to the additional remuneration for performing health hazard activities, that is, for those activities in which occupational risks persist.

Health hazards are associated with the causes of harms to health, as well as with activities and environments which, under specific conditions, expose workers to harmful agents, even if the damage that occurs is slight and imperceptible, as legally defined and classified in Annex 14 of NR 15 in force^(4)^.

According to article 190 of the current Consolidation of Labor Laws (*Consolidação das Leis do Trabalh*o - CLT)^(11)^, it is up to the Labor Department to approve the framework of health hazard activities and operations, the requirements and tolerance limits for characterizing the health hazard conditions of each of the agents that are harmful to health. Although they are ordinances arising from Regulatory Acts of the Executive Branch, they have normative force under the current legislation set out in article 200 of the CLT^(11)^ and in item XXII of article 7 of the Federal Constitution^(12-13)^.

However, this does not authorize *contra legis* legislation or silence on the biological risks experienced by Nursing professionals, given the different epidemiological scenario from the time the standard was elaborated and of restrictive and obtuse conditions, which offend the principle of human dignity.

According to data from FUNDACENTRO^(14)^, the various technical aspects of the Standard were discussed and elaborated by the then occupational hygiene technicians, without assembling a tripartite committee. With regard to the biological risks, Annex 14 of current NR 15^(4)^ lists activities that involve permanent contact with biological agents, whose health hazard is qualitatively characterized, with no evaluation of the intensity and time of exposure to biological agents, nor of the concentration of these agents in the environment.

After analyzing Annex 14^(4)^, inconsistencies are verified, namely: the assessment is qualitative and contact must be permanent, as there would be no way to define the exposure time for characterizing the biological risk. In addition to that, the focus is on the professional activity and not on the biological agents. Previously, the Safety Standards were provided for in various scattered administrative acts, with health hazard activities being listed in Table VII of MTPS (Ministry of Labor and Social Security *- Ministério do Trabalho e Previdência Social-MTPS*) Ordinance No. 491/1965^(6)^.

Subsequently, the theme was dealt with in Annex 14 of NR 15, established by MTb Ordinance No. 3,214/1978^(4)^, whose content was changed by SSMT (Safety and Occupational Medicine Secretariat - *Secretaria de Segurança e Medicina do Trabalho-SSMT*) Ordinance No. 12/79^(15)^. Table VII of Ordinance 491/65^(6)^ included, in the maximum degree of health hazard conditions, only work in contact with patients and infectious material in health institutions exclusively devoted to those isolated due to infectious diseases, such as sanatoriums for tuberculosis and leprosy patients. For the care of non-isolated patients and their infectious-contagious materials, that is, the other health activities, health hazard conditions were considered as average^(6)^.

However, the original version of Annex 14 of NR 15^(4,6)^ excluded the expression “isolation”, given the change in the profile of treatment sites for infectious diseases from 1960 onwards, which no longer required isolation of patients to avoid social exclusion, due to the scientific advances and to the existence of medications.

In addition to that, the text classified the Nursing professionals’ activity as of the highest degree, making no distinction between those who had contact with patients in isolation and the respective infectious-contagious material and the others^(6)^.

In 1979, Ordinance 12/79^(15)^, which is in force, once again brought the expression “isolation”, although without referring to exclusive institutions for this purpose. Under this aegis, the maximum degree becomes for professionals in contact with patients in isolation and the infectious-contagious material, in any hospital institution, and the medium degree corresponds to when there is contact with non-isolated patients or infectious material, in any other treatment site^(6)^.

The legal situation has remained the same since 1979: only the 40% additional is granted to the Nursing professionals who work with patients in isolation and the respective infectious materials, as an exception to the rule. As a general rule, the other health professionals are entitled to the 20% additional, regardless of their function.

It is evident that, as a simple observation, it is not fair that Nursing professionals, as a general rule, are subjected to 20% of the health hazard allowance due to the legal aspects imposed by Annex 14 of NR 15^(4)^, since the Standard is outdated not only in view of the current epidemiological scenario caused by the new coronavirus (SARS-CoV-2), but also by the existence of multi-resistant microorganisms, observed in the most diverse situations, which can cause illnesses to Nursing professionals, non-existent at the time when this normative was elaborated.

The situation has persisted since 1979, among other reasons, due to labor movement omission, as negligence in advocating the professional category and lack of sensitivity for this task have not received scrutiny of the Judiciary Branch. This reality, however, is not the same in other categories, as many questioned similar situations and demanded positioning of the Superior Labor Court, through Precedent 448^(16)^, namely:

“HEALTH HAZARD ACTIVITY. CARACTERIZATION. PROVISION IN REGULATORY STANDARD No. 15 OF MINISTRY OF LABOR ORDINANCE No. 3,214/78. SANITARY FACILITIES (conversion of Jurisprudential Guidance No. 4 of SBDI-1 - *Subseção I Especializada em Dissídios Individuais* - Sub-Section I Specialized in Individual Disputes - with new wording of item II) - Res. 194/2014, DEJT (*Diário Eletrônico da Justiça do Trabalho* - Electronic Diary of Labor Justice) disclosed on May 21^st^, 22^nd^ and 23^rd^, 2014. I - Verification of health hazard conditions by means of an expert report is not enough for the employee to be entitled to the respective additional, being necessary to classify the health hazard activity in the official list prepared by the Ministry of Labor. II - Cleaning of sanitary facilities for public or collective use of large circulation, and the respective garbage collection, for not equivalent to cleaning in homes and offices, gives rise to the payment of a maximum degree of health hazard allowance, subjected to the provisions of Annex 14 of NR 15 of MTE Ordinance No. 3,214/78 regarding collection and industrialization of urban waste”^(16)^.

Thus, the position of the Supreme Labor Court on the matter is clear since, if the activity of cleaning sanitary facilities for public use confers the worker maximum health hazard degree, Nursing professionals who work with bedridden patients, who need personal care with baths, use of urinals, acting directly with human excrement, in addition to other situations, are also entitled to have health hazard considered in its maximum degree. Thus, the Supreme Labor Court reiterates that the comparative reference has the sole scope of situating the problem under analysis.

To corroborate what is explained here, it is worth bringing to discussion the TST (*Tribunal Superior de Justiça* - Superior Court of Justice) decision^(17)^:

“(...) RECOGNIZED SOCIAL TRANSCENDENCE. HEALTH HAZARD ALLOWANCE. CLEANING COLLECTIVE BATHROOMS IN SCHOOLS. This is a request to condemn the payment of the health hazard allowance to its maximum degree, in which the plaintiff alleges that cleaning toilets and garbage collection were tasks inherent to her functions, which exposed her to contact with biological agents, being entitled to the payment of the referred allowance. The Regional Court states that, in accordance with Precedent 448, II, of the TST, situations that give rise to health hazard conditions ‘are only those in which sanitized bathrooms are open to the general population’. In this perspective, understanding that the plaintiff was only responsible for cleaning 1 toilet for collective use, used by nearly 240 students, it was concluded that the plaintiff is not entitled to the health hazard allowance. However, the position that has been adopted by this Superior Labor Court is in the sense that cleaning bathrooms for collective use, as in the case file, makes the payment of a health hazard allowance to a maximum degree due, as provided for in Annex 14 of NR 15 of the then MTE and jurisprudence based on Precedent 448, II, of the TST. Review appeal known and provided” by the TST, 2^nd^ Panel, Rapporteur Minister Delaide Miranda Arantes, DEJT 11/06/2020^(17)^.

Even cleaning toilets in schools entitles the highest degree of health hazard, which is not attributed to Nursing professionals. It becomes clear that this serious mistake must be reviewed.

Thus, NR 15^(4)^ is being silent and contrary to the current Federal Constitution, as it disregards the real epidemiological scenario of Nursing professionals and does not consider that the harms experienced by them is impossible to be removed, due to the very nature of the biological risk and of the health care work processes.

Therefore, the aforementioned Standard is not compatible and is applied to the detriment of the health of countless professionals, who suffer changes in their natural microbiota and drug resistance, due to exposure to biological agents, as well as risk of life due to the SARS-CoV-2 virus and its mutations. It is therefore a real offense to human dignity.

### The principle of human dignity: Constitutional foundation of the health hazard allowance

The Federative Republic of Brazil is based on the assumption of human dignity, as provided for in item III of article 1 of the current FC^(13)^ thus permeating all existing relations in the country. In this sense, the constituent, by enshrining human dignity as one of the foundations of the Democratic State of Law, recognized that the State exists in function of the human person and this constitutes the main purpose and not the means of State activity^(18)^.

There is no consensual and universal definition on the theme of dignity, as it is an intrinsic quality of the human being. This makes it worthy of respect and consideration, both by the State and by the community, implying a complex of fundamental rights and duties that ensure minimum existential conditions for a healthy life, protection against any inhuman or degrading act and active and co-responsible participation in one’s own existence, as well as in the relationships with other human beings^(19)^.

The constituent, by providing that human dignity underlies the Democratic State of Law, proclaimed that, in concrete and everyday cases, when there is a gap between the circumstances surrounding human life, the impasses must be resolved with the effectiveness of the Constitutional Rules, the application of the law and the State’s obligation to provide positive benefits^(20)^.

In addition to that, the Federative Republic of Brazil indicates commitments and ideals in its constitutional preamble. Thus, it aims at establishing a Democratic State based on social and individual rights, freedom, security, well-being, development, equality and justice. Such values are paramount in a fraternal, pluralistic, supporting and unprejudiced society^(21)^.

Therefore, human dignity is a supreme value that attracts the content of all fundamental rights. It is a concept that requires densification of values, in order not only to reduce its meaning in terms of defending traditional personal rights, but also to invoke social rights, guaranteeing the basis of human existence^(20)^.

After analyzing the Brazilian state constitutions, it is verified that there is multiplicity of associations between the principle of human dignity and the fundamental rights, emphasizing that this principle is the starting point for other rights^(18)^.

Thus, as stated in Articles 170 and 205 of the Federal Constitution^(13)^, the economic order aims at ensuring the dignified existence of education, personal development and preparation for the exercise of citizenship, as well as compensation for the performance of an activity work in an unhealthy environment, among others, not as mere formal statements, but as indicators of the effective normative content of human dignity.

The principle of human dignity sometimes appears as one of personality and others as a principle of individuality, which concerns a commitment to absolute and unrestricted respect for the identity and integrity of every human being as a subject of rights^(20)^. Consequently, the worker’s physical integrity must be protected. If workers are exposed to a health hazard environment, which cannot be mitigated or excluded by the use of Personal Protective Equipment (PPE), they must have guaranteed the right to fair compensation for health harms suffered due to exposure to biological agents, as the last reason of the corollary of human dignity protection.

It is true that these provisions are for the protection of the worker’s life and health. Thus, there should initially be strive for a safe and healthy work environment, with elimination of the unhealthy agents and of all the risks of accidents. However, given the impossibility of predicting all situations, in a complex society and in a labor complex, it is commendable that these risks, when not eliminated, are minimized through the use of PPE. However, the statistics on occupational accidents and diseases are evident and constant, with or without compliance with the rules for the protection of health and safety in the work environment, which indicates that the protection offered by individual equipment must not be fully relied upon.

Even if not entering into the discussion of the monetization of health and life, which is strongly fought against, it is imperative to understand that health hazard conditions are due, but in a fair way, if it is possible to say that this allowance compensates for the risk to health and life. This principle has supreme value, attracting the content of all fundamental human rights, as it consolidates the strength of other rights and, for this very reason, the principle of human dignity is also the foundation of the right to the health hazard allowance, which stands in line with the principle of the prohibition of insufficient protection.

Compensation must be equivalent to the harms incurred to the workers’ health, for working in risk environments. It also has two dimensions: a negative one, referring to the fact that the person cannot be object of offenses or humiliation; and a positive one, in the sense of protecting the full development of the personality, which is infringed due to non-compliance with safety and health standards of the worker.

In the case of Nursing professionals, the Regulatory Standard is obsolete and inadequate to the biological risks borne by Nursing, given that it was elaborated in an epidemiological context different from the one currently experienced by these workers^(6)^.

The Federal Constitution of 1988^(12)^ linked infra-constitutional normativity to a principle-based framework, resulting in the fact that any new amendments to its text or infra-constitutional legislation should be involved by these principles. Thus, Annex 14 of NR 15^(4)^, which has normative force, must also be involved in these principles. Therefore, the Standard must compensate workers for exposure to the biological agent in an effective and consistent manner with the working conditions faced and not define an illegal and unfair percentage.

Parallel to this, it is imperious to ask whether this provision of normative setting of the percentage of unhealthy conditions would have been accepted by FC/88^(13)^. It is understood that no, although this fact has not yet been object of judicial questioning. By establishing human dignity as the foundation of the Democratic State of Law, the constituent authorized the interference of this principle throughout the constitutional body, thus offering a hermeneutical guideline of extension throughout the field of the legal order.

Thus, it is appropriate to protect the physical integrity of human beings in the individual dimension, as well as spiritual integrity with regard to their subjectivity. As a value-fundamental, human dignity does not represent only a hermeneutics principle, but the reason for the existence of the Constitution^(20)^. For this reason, its concept is dynamic and cannot be restricted to an obsolete normative provision, which does not contemplate such a requirement.

Human dignity has absolute value, attracting all fundamental rights. In this context, it is understood that it is perfectly applicable to the workers as a unifying value of the right to life, which is broken down into the right to physical integrity. That said, since the exposure to biological agents of these professionals cannot be excluded or mitigated with the use of PPE, there is the right to compensation through the health hazard allowance, in a degree consistent with the risk of their exposure.

Therefore, the Nursing professionals’ health hazard allowance due to exposure to biological agents cannot be classified as a mere normative framework. There must be effective observance of the biological agents to which these workers are exposed, such as SARS-CoV-2.

It is therefore inferred that the impossibility of measuring the time of exposure to the agents causing unhealthy conditions offends the principle of human dignity, as well as the legislation on the subject matter, in a perspective of general interpretation of the institute, which makes the legal provision totally unconstitutional.

In view of this, it is necessary to adapt the standards relating to workers’ safety and health, through the effective participation of the interested spheres, namely: workers, employers and government. Such standards must have human dignity as a guiding bias, as a corollary of the last reason (*ratio*), in line with the protection of the Nursing professionals’ life and health.

## Conclusion

Given what has been elucidated here, it is concluded that it is necessary to broaden the discussion on the theme and to review the percentage of the unhealthy work additional for Nursing professionals due to exposure to potentially contaminating biological agents in their work environments. Such purpose aims at granting a fair compensation to Nursing professionals, either to confer the maximum degree of the additional, based on legal and occupational parameters, or to grant the legitimate right of the Nursing professional to the technical proof of health hazard conditions of their working environment, since the health hazard allowance is a worker’s right and is based on human dignity. Thus, it is imperative and urgent to mobilize jurists, public policy managers, the Federal Council of Nursing, Universities and Brazilian Nursing professionals to provide fair compensation to Nursing professionals due to exposure to potentially contaminated biological agents in their work environments.

## Footnotes

* Fair, in the sense of equal treatment. Treating the equal equally and the different differently in proportion to their inequalities, but not creating inequality by mere elective criterion.

## References

[1] Silva RN, Ferreira MA (2021). Nursing and society: Evolution of Nursing and of capitalism in
the 200 years of Florence Nightingale. Rev. Latino-Am. Enfermagem. [Internet].

[2] Porto JS, Marziale MHP (2020). Construction and validation of an educational video for improving
adherence of nursing professionals to standard precautions. Texto Contexto Enferm. [Internet].

[3] Organização das Nações Unidas (2015). Agenda 2030 para o desenvolvimento sustentável. [Internet].

[4] Ministério da Economia (BR) (1978). Norma Regulamentadora n.º 15. [Internet].

[5] Nichiata LYI, Gir E, Takahashi RF, Ciosak SI (2004). Evolution of the isolation of contagious diseases: knowledge in
contemporary practice. Rev Esc Enferm USP [Internet].

[6] Ministério da Economia (BR) (2019). Estudo Técnico - Anexo 14 da Norma Regulamentadora n.º 15 - Agentes
Biológicos. [Internet].

[7] Curtis VA (2007). Dirt, disgust and disease: a natural history of
hygiene. J Epidemiol Community Health. [Internet].

[8] Czeresnia D (1997). Do contágio à transmissão: ciência e cultura na gênese do
conhecimento epidemiológico. Rio de Janeiro: Editora FIOCRUZ.

[9] Schramm JMA, Oliveira AF, Leite IC, Valente JG, Gadelha AMJ, Portela MC (2004). Epidemiological transition and the study of burden of disease in
Brazil. Ciênc Saúde Coletiva. [Internet].

[10] Fracarolli IFL, Oliveira SA, Marziale MHP (2017). Bacterial colonization and antimicrobial resistance in healthcare
workers: an integrative review. Acta Paul Enferm. [Internet].

[11] Presidência da República (BR), Casa Civil, Subchefia para Assuntos
Jurídicos (1943). Decreto-lei n.º 5.452, de 1º de maio de 1943. Aprova a Consolidação das
Leis do Trabalho. [Internet].

[12] Belmonte AA, Martinez L, Maranhão N (2020). O Direito do Trabalho na crise da COVID-19.

[13] Brasil (2016). Constituição da República Federativa do Brasil [Internet].

[14] Ministério da Economia (BR) (2020). Subsecretaria de Inspeção do Trabalho. [Internet].

[15] Ministério do Trabalho e Emprego (BR) (1979). Portaria SSST n.º 12, de 12 de novembro de 1979. [Internet].

[16] Poder Judiciário (BR), Justiça do Trabalho, Tribunal Superior do
Trabalho (2014). Converte a Orientação Jurisprudencial n.º 4 da SBDI-I com nova redação
do item II. [Internet].

[17] Poder Judiciário (BR), Justiça do Trabalho, Tribunal Superior do
Trabalho (2020). Processo: RR-10957-19.2017.5.03.0014. [Internet].

[18] Mendes GF (2013). A dignidade da pessoa humana na Constituição Federal de 1988 e
sua aplicação pelo Supremo Tribunal Federal. Observatório da Jurisdição Constitucional. [Internet].

[19] Sarlet IW (2004). A eficácia dos direitos fundamentais na Constituição Federal de
1988.

[20] Lora APJ (2004). Patrimônio genético humano e sua proteção na Constituição Federal de
1988.

[21] Araujo LAD, Nunes VS (2003). Curso de direito constitucional.

